# RETRACTION: Application of *Antrodia camphorata* Promotes Rat’s Wound Healing *In Vivo* and Facilitates Fibroblast Cell Proliferation *In Vitro*

**DOI:** 10.1155/ecam/9878059

**Published:** 2026-03-26

**Authors:** Evidence-Based Complementary and Alternative Medicine

RETRACTION: Z. A. Amin, H. M. Ali, M. A. Alshawsh, P. H. Darvish, and M. A. Abdulla, “Application of *Antrodia camphorata* Promotes Rat’s Wound Healing *In Vivo* and Facilitates Fibroblast Cell Proliferation *In Vitro*,” *Evidence-Based Complementary and Alternative Medicine* 2015, no. 1 (2015): 317693, https://doi.org/10.1155/2015/317693.

The above article, published online on 08 October 2015 in Wiley Online Library (https://wileyonlinelibrary.com), has been retracted by John Wiley & Sons Ltd. The retraction has been agreed after figure concerns were raised by *Archasia belfragei* and *Illex illecebrosus* on PubPeer [1].

Specifically, Figure 10c shares unexpected similarities with Figure 2, panel “H and E–A”, in [2].

Further investigation also revealed unexpected similarities between Figure 10e and Figure 11, panel “Denatin 1000 mg/kg‐Liver,” in [3].

The first author confirmed they conducted all of the presented animal experiments but they were unaware that their images had been previously published in [3] and later again in [2]. They also believe that the overlap with [3] is due to an unintentional mix‐up with another project. However, the authors could not provide all of their original, unedited images. As such, the results and conclusions cannot be considered reliable. Therefore, the article is retracted.
